# Effect of PLA-g-MAH Content on the Interfacial Structure and Properties of Wheat Straw/PLA Biocomposites

**DOI:** 10.3390/polym18182245

**Published:** 2026-09-15

**Authors:** Yuqing Wang, Shilong Zheng, Fangmin Yuan, S. Siti Suhaily

**Affiliations:** 1 School of the Arts, Universiti Sains Malaysia (USM), Minden 11800, Penang, Malaysia; 2 School of Civil Engineering, Hebei University of Science and Technology, Shijiazhuang 050018, China; 3 Nusantara Institute of Creative Arts (INSAN), Universiti Teknologi MARA (UiTM), Shah Alam 40450, Malaysia

**Keywords:** wheat straw, polylactic acid, PLA-g-MAH, biocomposites, agricultural residues, natural fibre composites, mechanical properties, water absorption

## Abstract

Wheat straw/poly(lactic acid) (WS/PLA) biocomposites provide a promising route for agricultural residue valorization, but poor interfacial compatibility limits their performance. This study investigated the effect of maleic-anhydride-grafted PLA (PLA-g-MAH) on WS/PLA biocomposites containing 50 wt% wheat straw. PLA-g-MAH was incorporated at 0–10 wt% of the total composite mass by replacing an equivalent proportion of neat PLA, while the wheat straw content was maintained at 50 wt%. The composites were evaluated through mechanical testing, density and water absorption measurements, X-ray diffraction (XRD), scanning electron microscopy (SEM), thermogravimetric analysis (TGA), and differential scanning calorimetry (DSC). Mechanical properties improved with increasing compatibilizer content and then declined at excessive dosages. At 7 wt% PLA-g-MAH, tensile and impact strengths reached 17.24 MPa and 15.56 kJ/m^2^, increasing by approximately 72% and 55%, respectively, compared with the uncompatibilized control. Density increased to 1.22 g/cm^3^, while water absorption decreased by about 43.2%. XRD indicated that moderate PLA-g-MAH addition promoted more favorable crystallization, whereas excessive addition reduced peak intensity and broadened diffraction peaks. SEM showed that 7 wt% PLA-g-MAH produced the most compact fibre–matrix interface, with fewer voids and less fibre pull-out. Thermal analyses further confirmed changes in thermal stability and crystallization behaviour after PLA-g-MAH addition. Overall, approximately 7 wt% PLA-g-MAH provided the best balance of interfacial integrity, mechanical performance, and moisture resistance.

## 1. Introduction

Natural fibre-reinforced biodegradable polymer composites have received increasing attention as potential alternatives to conventional petroleum-based composite materials because they combine renewable lignocellulosic resources with biodegradable polymer matrices [1,2,3]. Among the available bio-based polymers, poly(lactic acid) (PLA) is widely used because of its good processability, relatively high stiffness, and commercial availability [4,5]. Agricultural residues such as wheat straw are also attractive as reinforcing materials because they are abundant, renewable, and rich in cellulose, hemicellulose, and lignin [6,7,8]. Developing cost-effective and biodegradable engineering materials is essential for enhancing industrial eco-efficiency and promoting sustainable development [9,10]. Incorporating wheat straw into PLA therefore provides a potential pathway for converting low-value agricultural residues into higher-value biocomposite materials. However, the performance of wheat straw/PLA (WS/PLA) composites is strongly dependent on the quality of the fibre-matrix interface [11].

A major challenge in WS/PLA composites arises from the different surface characteristics of the two components. Wheat straw contains abundant hydroxyl groups associated with cellulose and hemicellulose and consequently exhibits relatively hydrophilic behaviour, whereas PLA is comparatively hydrophobic [12,13]. This mismatch in polarity and surface energy can lead to insufficient wetting of wheat straw by the PLA matrix, weak interfacial adhesion, interfacial voids, fibre pull-out, and local stress concentrations [14,15,16]. These defects reduce the efficiency of stress transfer between the reinforcing phase and the polymer matrix and can adversely affect both mechanical performance and resistance to moisture penetration [17]. Therefore, improving fibre–matrix compatibility is essential for obtaining WS/PLA biocomposites with more stable and balanced engineering properties.

Compatibilization is one of the most widely adopted approaches for improving the interface of natural fibre/polymer composites. Maleic-anhydride-grafted poly(lactic acid) (PLA-g-MAH) is particularly suitable for PLA-based systems because its molecular structure contains both reactive maleic anhydride groups and PLA chain segments [18,19]. The maleic anhydride groups can interact with hydroxyl-containing sites on lignocellulosic fibres through possible esterification and hydrogen-bonding interactions, whereas the PLA segments are compatible with the surrounding PLA matrix [20]. PLA-g-MAH can therefore act as an interfacial bridge between the wheat straw reinforcement and PLA matrix, promoting fibre wetting and more efficient load transfer. Previous studies have demonstrated that the effectiveness of maleated compatibilizers in natural-fibre/PLA systems is strongly dependent on formulation and processing conditions. Nyambo et al. reported that the addition of 3–5 phr PLA-g-MA to PLA/wheat-straw composites increased tensile and flexural strengths by approximately 20% and 14%, respectively [21]. In wood-fibre/PLA composites, Zhang et al. found that the tensile strength reached 47.41 MPa at 30 wt% MAH-g-PLA, corresponding to an approximately 9% improvement over the uncompatibilized composite [22]. However, the beneficial effect is not universal. Ucpinar et al. reported that the incorporation of 1–5 wt% MAH-g-PLA into PLA/PBS/hemp-fibre composites did not produce a considerable improvement in tensile strength, despite changes in crystallinity and thermomechanical behaviour [18]. These contrasting findings indicate that compatibilizer efficiency and optimum dosage depend on fibre chemistry, matrix composition, fibre loading, and processing conditions, and therefore cannot be directly generalized across different natural-fibre/PLA systems.

However, the beneficial effect of PLA-g-MAH is not necessarily proportional to its concentration. An insufficient amount of compatibilizer may provide limited interfacial coverage and inadequate interaction with available hydroxyl-containing fibre surfaces, whereas excessive PLA-g-MAH may accumulate within the interfacial region or form locally enriched phases [23]. Such excess compatibilizer may also produce a plasticization- or dilution-like effect, thereby reducing the structural uniformity of the composite and weakening the improvements obtained at moderate compatibilizer levels [18]. The experimental observations in WS/PLA systems therefore suggest the existence of an optimum compatibilizer concentration rather than a continuously increasing performance response [21,24]. In particular, changes in mechanical properties should be considered together with interfacial morphology, crystallization behaviour, internal compactness, and moisture resistance in order to understand the overall effect of PLA-g-MAH.

Although the compatibilizing effect of maleic-anhydride-grafted polymers has been investigated in various natural fibre/polymer systems, the relationship between PLA-g-MAH dosage and the combined mechanical, physical, crystalline, and microstructural responses of high-content WS/PLA biocomposites remains insufficiently clarified [22]. Many studies focus primarily on one or two macroscopic performance indicators, while less attention has been given to establishing a dosage-dependent structure–property relationship by integrating mechanical testing with density, water absorption, X-ray diffraction (XRD), and scanning electron microscopy (SEM) [25]. Such an integrated assessment is particularly important for composites containing a relatively high lignocellulosic fraction, where interfacial defects can substantially influence both load transfer and moisture transport [25,26]. The novelty of the present study lies in the dosage-resolved evaluation of PLA-g-MAH in a high-wheat-straw-loading (50 wt%) PLA system, integrating mechanical performance, density, water absorption, XRD, SEM, and thermal characterization to establish a unified structure–property relationship and identify an optimum compatibilizer content.

Accordingly, the present study investigated the effect of PLA-g-MAH content on WS/PLA biocomposites containing a fixed wheat straw fraction of 50 wt%. PLA-g-MAH was introduced over a range from the uncompatibilized control to 10 wt%, and its influence was evaluated through tensile, flexural, and impact testing, density and water-absorption measurements, XRD analysis, SEM observation, TGA, and DSC. The experimental design in the study specifically examined different PLA-g-MAH dosages while maintaining the wheat straw content at 50 wt%. The objectives were to (1) determine the dosage-dependent effect of PLA-g-MAH on the mechanical and physical properties of WS/PLA biocomposites; (2) clarify the relationship between compatibilizer content, crystalline and thermal behaviour, and fibre–matrix interfacial morphology; and (3) identify an appropriate PLA-g-MAH concentration that provides a balanced improvement in mechanical performance, internal structural integrity, and moisture resistance.

## 2. Materials and Methods

### 2.1. Raw Materials

Wheat straw (WS) without wheat ears was obtained from Shijiazhuang, Hebei Province, China. Before use, the wheat straw was cleaned to remove adhering dust and impurities and subsequently cut and ground for composite preparation. Apart from cleaning and mechanical size reduction, no additional chemical or surface pretreatment was applied to the wheat straw in the present PLA-g-MAH compatibilization experiment.

Poly(lactic acid) (PLA, grade 4032D) was supplied by NatureWorks, LLC, Plymouth, MN, USA. According to the manufacturer’s technical specifications, PLA 4032D has a specific gravity of approximately 1.24 and a melt flow rate of approximately 7 g/10 min at 210 °C under a load of 2.16 kg.

Maleic-anhydride-grafted poly(lactic acid) (PLA-g-MAH), supplied as a white solid, was obtained from Sinopharm Chemical Reagent Co., Ltd., Shanghai, China. According to the supplier’s technical specifications, the PLA-g-MAH had a maleic anhydride grafting degree of 2.0–4.0%, determined by acid–base titration, a melt flow rate of 3.0–6.0 g/10 min at 190 °C under a load of 2.16 kg, and a density of 1.20 g/cm^3^. The reported loss on drying was ≤0.3% at 105 °C for 10 min, and the melting temperature was approximately 120 °C, determined according to ASTM D3418 [27].

Polytetrafluoroethylene (PTFE) film was used during hot pressing to prevent adhesion between the composite and the mould surfaces [28]. Before composite preparation, WS, PLA, and PLA-g-MAH were dried at 80 °C for 24 h and subsequently stored in a desiccator to minimize moisture reabsorption. This pretreatment was performed to reduce moisture-related defects and minimize hydrolytic degradation of PLA during hot pressing [29].

### 2.2. Preparation of PLA-g-MAH-Compatibilized WS/PLA Biocomposites

The wheat straw content was maintained at 50 wt% in all formulations. PLA-g-MAH was incorporated at 0–10 wt% of the total composite mass by replacing an equivalent amount of neat PLA. Accordingly, as the PLA-g-MAH content increased, the neat PLA content decreased while the total composite mass and wheat straw fraction remained constant. The investigated PLA-g-MAH contents were 0, 5, 6, 7, 8, 9, and 10 wt%, with the 0 wt% formulation serving as the uncompatibilized control. The detailed formulations are summarized in Table 1.

For each batch, the total mass of the formulation was maintained at 8 g. The required amounts of dried WS, PLA, and PLA-g-MAH were weighed using an analytical balance with an accuracy of ±0.01 g and thoroughly dry-mixed to obtain a homogeneous blend. The mixture was then evenly distributed in a preheated hot-press mould, with PTFE films placed on both surfaces to prevent sticking. Hot pressing was conducted at 180 °C and 14 MPa for 20 min. After hot pressing, the mould was cooled naturally to room temperature before demoulding. The resulting composite panels had dimensions of 80 mm × 80 mm × 2 mm and were subsequently cut into specimens for mechanical, physical, and characterization tests.

The hot-pressing parameters (180 °C, 14 MPa, and 20 min) were adopted from our previous process-optimization study on hot-pressed wheat-straw/PLA biocomposite boards, in which an L9 (3^3^) orthogonal design was used to screen the processing conditions and this parameter combination was subsequently selected for composite preparation [30]. Because prolonged exposure of PLA to the molten state may promote thermal and hydrolytic chain degradation, all raw materials were dried at 80 °C for 24 h and stored in a desiccator before processing to minimize moisture-induced hydrolysis. Nevertheless, molecular-weight changes during hot pressing were not directly quantified in the present study, and possible processing-induced degradation cannot be completely excluded.

### 2.3. Mechanical Testing

**Figure 1:**
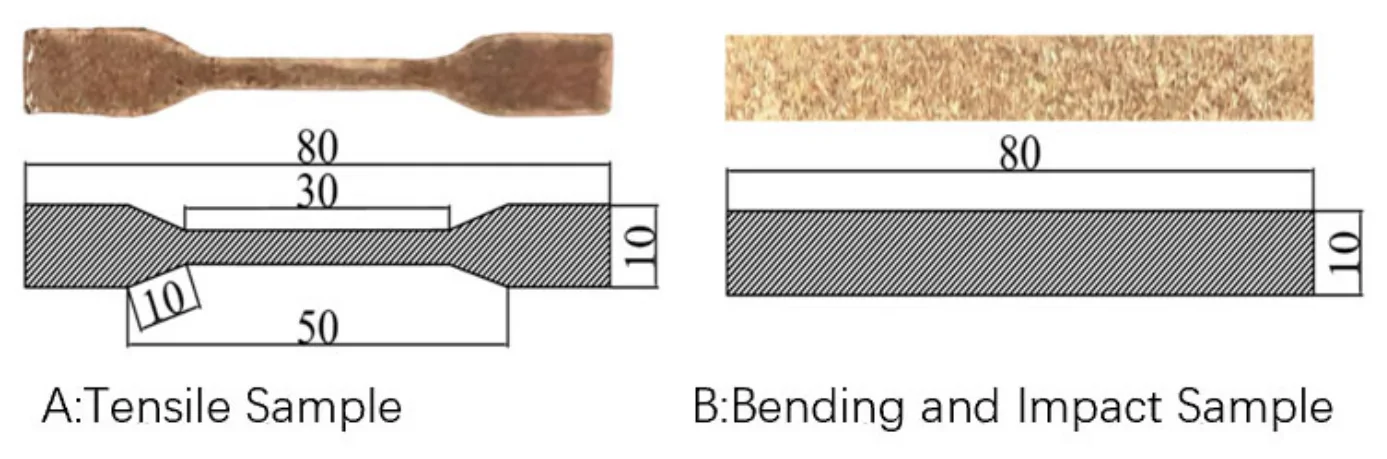
Geometry and dimensions of specimens used for mechanical testing: (**A**) tensile specimen; (**B**) bending and impact specimens. All dimensions are in mm, and the specimen thickness was 2 mm.

The mechanical performance of the WS/PLA biocomposites was evaluated in terms of flexural, impact, and tensile properties. Five specimens were tested for each condition, and the average value was reported. The tests were conducted according to the corresponding Chinese national standards used in the experiment. Mechanical test specimens were cut directly from the hot-pressed composite panels. The tensile specimens had a dog-bone geometry, whereas the bending and impact specimens were rectangular bars with dimensions of 80 mm × 10 mm × 2 mm. The specimen geometries and detailed dimensions are shown in Figure 1.

(a) Bending strength test

The bending test was conducted on a universal testing machine in accordance with GB/T 1449-2005, Fibre-reinforced plastic composites-Determination of flexural properties [31]. Rectangular test bars were subjected to three-point bending with a span L = 60 mm and a crosshead speed of 2 mm/min. Each bar was placed flat on the two supports with the loading nose centred. The machine was activated, and a load was applied until fracture; the maximum load P was recorded. Five specimens were tested, and the average value was reported. Bending strength Sigma with subscript f was calculated by:

(1) $$\sigma_{f}=\frac{3PL}{2bd^{2}}$$

where P is the maximum load, *L* is the span, *b* is the width, and *d* is the thickness.

(b) Impact strength test

The impact strength was measured in accordance with GB/T 1043-2008, Plastics-Determination of Charpy impact properties, using a simply supported beam impact tester (PIT-J/PIT501J) [32]. The impact specimens were identical to the bending bars. Each bar was positioned in the fixture with the notched face facing the striker. The hammer was released to strike the centre of the specimen, and the energy E absorbed. Five specimens were tested, and the average value was reported.

Impact strength was calculated as:

(2) $$\alpha_{3}=\frac{E}{bd}\times 10^{3}$$

where α_3_ is specimen impact strength (kJ/m^2^); E is impact energy absorbed by the sample (J); b is specimen width (mm); and d is sample thickness (mm).

(c) Tensile strength test

The tensile test was performed on a universal testing machine in accordance with GB/T 1040.2-2006, Plastics-Determination of tensile properties-Part 2: Test conditions for moulding and extrusion plastics [33]. The test was conducted at a rate of 5 mm/min. The specimen ends were secured in the grips, and load was applied evenly until fracture occurred. The maximum tensile load was recorded. Five specimens were tested, and the average value was calculated.

(3) $$\alpha_{1}=\frac{P}{bd}$$

where a_1_ is the tensile strength of the specimen (MPa), P is the maximum tensile load (N) that the specimen can withstand, b is the specimen width (mm), and d is the sample thickness (mm).

### 2.4. Physical Property Testing

#### 2.4.1. Density Measurement

Density was determined using the Archimedes displacement method according to GB/T 1033.1-2008. Before testing, the specimens were dried at 60 °C for 4 h. Their masses in air and in deionized water at 25 ± 1 °C were subsequently recorded using an analytical balance with an accuracy of 0.001 g. Three specimens were tested for each formulation, and the average density was calculated.

Density was calculated as:

(4) $$\rho =\frac{m_{1}}{m_{1}-m_{2}}\rho_{w}$$

where $m_{1}$ is the mass of the specimen in air, $m_{2}$ is the apparent mass in water, and $\rho_{w}$ is the density of water at the testing temperature (0.997 g/cm^3^ at 25 °C).

#### 2.4.2. Water Absorption Test

Water absorption was determined according to GB/T 1034-2008 [34]. The specimens were first dried at 105 °C for 24 h and weighed to obtain their initial dry mass. They were then fully immersed in deionized water at 25 ± 1 °C for a total of 120 h. Water absorption was measured after 24, 48, 72, 96, and 120 h of immersion. At each time point, the specimens were removed from the water, surface moisture was gently removed with filter paper, and the specimens were immediately weighed. They were then re-immersed in deionized water until the next measurement. Three replicate specimens were tested for each formulation.

Water absorption was calculated as:

(5) $$WA\left(\%\right)=\frac{m_{w}-m_{d}}{m_{d}}\times 100$$

where $m_{d}$ is the initial dry mass and $m_{w}$ is the specimen mass after water immersion.

### 2.5. X-Ray Diffraction Analysis Characterization

X-ray diffraction (XRD) was employed to examine changes in the crystalline structure of the WS/PLA composites associated with PLA-g-MAH addition. Measurements were conducted using Cu Kα radiation with a wavelength of 0.15418 nm. The operating voltage and current were 40 kV and 30 mA, respectively. The scanning range was $2\theta =5^{\circ }-40^{\circ }$, with a scanning rate of 2°/min and a step size of 0.02°. Changes in diffraction peak position, intensity, and width were analyzed to assess differences in crystallization behaviour among the formulations.

### 2.6. Scanning Electron Microscopy

Scanning electron microscopy (SEM; JSM-IT300, JEOL Ltd., Akishima, Tokyo, Japan) was used to examine the fracture morphology and fibre–matrix interface of the WS/PLA composites. Fracture surfaces were prepared by liquid-nitrogen embrittlement and sputter-coated with an approximately 10 nm thick gold layer before observation. SEM observations were conducted at an accelerating voltage of 10 kV and magnifications ranging from 200× to 5000×. Particular attention was given to fibre dispersion, interfacial gaps, fibre pull-out, matrix adhesion, pore distribution, and fracture characteristics.

SEM observations were subsequently interpreted together with the mechanical and physical results to assess changes in interfacial integrity associated with PLA-g-MAH addition.

### 2.7. Thermal Analysis

#### 2.7.1. Thermogravimetric Analysis

Thermogravimetric analysis (TGA) was performed to evaluate the thermal stability and degradation behaviour of WS/PLA biocomposites containing different PLA-g-MAH contents. The samples were heated from room temperature to 700 °C at a heating rate of 10 °C min^−1^ under a nitrogen atmosphere with a flow rate of 50 mL min^−1^. The residual mass was continuously recorded as a function of temperature. Derivative thermogravimetric (DTG) curves were obtained from the first derivative of the TG data. The onset degradation temperature $T_{onset}$, maximum degradation temperature $T_{max}$ and residual mass were used to evaluate the thermal stability of the composites.

#### 2.7.2. Differential Scanning Calorimetry

Differential scanning calorimetry (DSC) was performed to investigate the melting and crystallization behaviour of the WS/PLA biocomposites containing different PLA-g-MAH contents. The samples were first heated from 30 to 210 °C at a heating rate of 10 °C min^−1^ and maintained at 210 °C for 5 min to eliminate the previous thermal history. Subsequently, the samples were cooled to −20 °C at a cooling rate of 20 °C min^−1^. During the second heating cycle, the samples were reheated from −20 to 210 °C at a heating rate of 10 °C min^−1^. The crystallization temperature (Tc) was determined from the cooling curves, whereas the glass transition temperature (Tg), cold crystallization temperature (Tcc), and melting temperature (Tm) were obtained from the second-heating curves. The melting enthalpy (ΔHm) and cold-crystallization enthalpy (ΔHcc) were used to calculate the degree of crystallinity (Xc) of the PLA phase according to the following equation:

(6) $$X_{c}(\%)=\frac{\Delta H_{m}-\Delta H_{cc}}{w_{\mathrm{PLA}}\Delta H_{m}^{0}}\times 100$$

where *Xc* is the degree of crystallinity of PLA, Δ*H_m_* is the melting enthalpy, Δ*H_cc_* is the cold-crystallization enthalpy, $w_{PLA}$ is the mass fraction of PLA in the composite, and ${\Delta H}_{m}^{0}$ is the melting enthalpy of 100% crystalline PLA, taken as 93.7 J g^−1^.

### 2.8. Statistical Analysis

Experimental data were analyzed using SPSS 26.0, while OriginPro 2022 was used for graphical presentation. Descriptive statistics were calculated for the mechanical and physical properties. Normality and homogeneity of variance were assessed before inferential analysis. Analysis of variance (ANOVA) was used to evaluate differences among PLA-g-MAH content groups, followed by Tukey’s HSD post hoc test for multiple comparisons when significant differences were detected. A value of *p* < 0.05 was considered statistically significant.

## 3. Results and Discussion

### 3.1. Mechanical Properties

**Figure 2:**
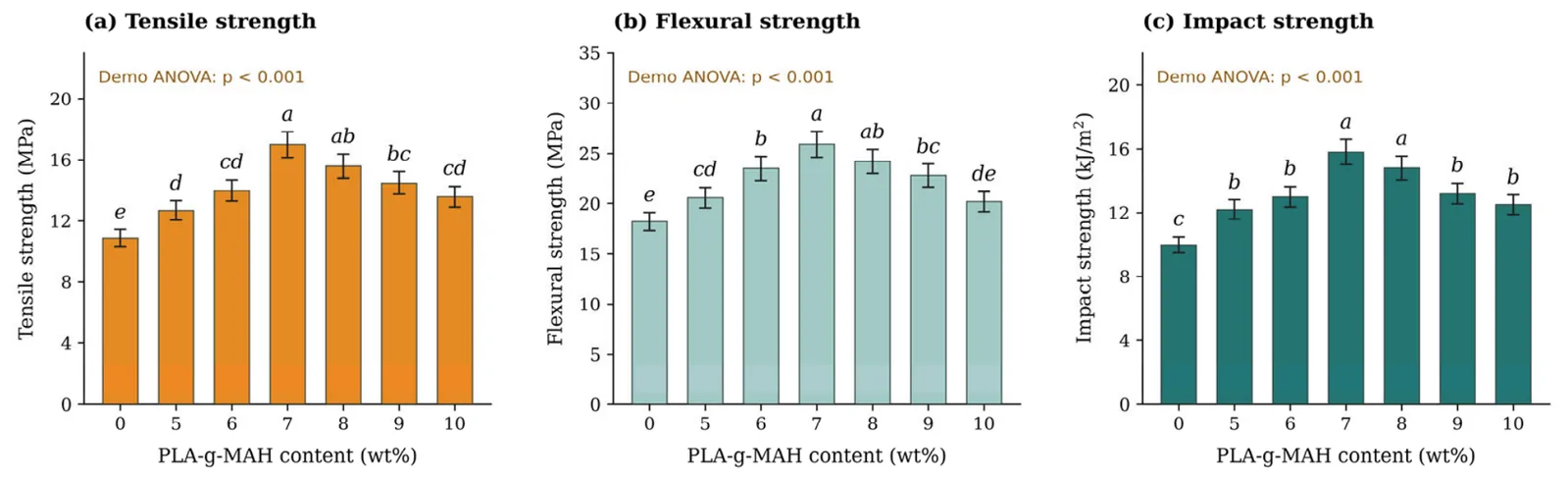
Effect of PLA-g-MAH content on the tensile, flexural, and impact properties of WS/PLA biocomposites. Data are presented as mean ± SD (n = 5). Different lowercase letters indicate significant differences among PLA-g-MAH content groups, whereas groups sharing the same letter are not significantly different, according to Tukey’s HSD post-hoc test (*p* < 0.05).

The effect of PLA-g-MAH content on the mechanical properties of the WS/PLA biocomposites is shown in Figure 2. Overall, the tensile, flexural, and impact responses exhibited a non-monotonic dependence on compatibilizer dosage, with the mechanical performance improving at moderate PLA-g-MAH contents and declining when the compatibilizer was added in excess. The most favorable overall mechanical response was observed at approximately 7 wt% PLA-g-MAH.

The tensile strength increased from 10.00 MPa for the uncompatibilized control to 17.24 MPa at 7 wt% PLA-g-MAH, corresponding to an increase of approximately 72%. The impact strength showed a similar response, increasing from 10.00 to 15.56 kJ/m^2^, an improvement of approximately 55%. The flexural strength followed the same general dose-dependent trend, reaching its highest region at approximately 7 wt% PLA-g-MAH before decreasing at higher compatibilizer contents.

As summarized in Table 2, density and mechanical strength followed similar trends with increasing PLA-g-MAH content. The 7 wt% formulation exhibited the highest density of 1.22 g/cm^3^, together with tensile, flexural, and impact strengths of 17.0 MPa, 25.9 MPa, and 15.8 kJ/m^2^, respectively. These consistent trends, considered alongside the SEM observations, suggest that improved interfacial compactness contributes to more effective load transfer. Further addition reduced both density and strength. However, formulations with similar densities, such as those containing 5 and 9 wt% PLA-g-MAH, exhibited different mechanical strengths, suggesting that density alone may not fully explain the mechanical response and that interfacial adhesion and structural uniformity also contribute.

The improvement in mechanical performance at moderate PLA-g-MAH contents can primarily be attributed to enhanced fibre–matrix compatibility. Without compatibilizer, the polarity difference between the hydrophilic wheat straw surface and the PLA matrix results in weak adhesion and facilitates interfacial debonding. PLA-g-MAH contains polar maleic anhydride groups that can interact with hydroxyl-containing sites on the lignocellulosic fibre surface, while its PLA backbone is compatible with the surrounding PLA matrix [35]. Consequently, PLA-g-MAH can promote stronger interfacial interactions and more efficient stress transfer across the fibre–matrix interface. The proposed interfacial-bridge mechanism is consistent with the molecular rationale used in the original experimental design.

The decline in mechanical properties above approximately 7 wt% indicates that the compatibilization effect reaches an optimum rather than increasing continuously with dosage. At 8–10 wt%, excess PLA-g-MAH may accumulate locally in the interfacial region or contribute to a comparatively softer compatibilizer-rich phase. Such heterogeneity can reduce effective stress transfer and facilitate local deformation or crack initiation. The mechanical deterioration observed at excessive PLA-g-MAH contents is therefore consistent with an over-compatibilization effect rather than insufficient interfacial modification.

Taken together, these results indicate that approximately 7 wt% PLA-g-MAH provides the most favorable balance between interfacial reinforcement and preservation of matrix load-bearing capacity in the present WS/PLA system.

**Figure 3:**
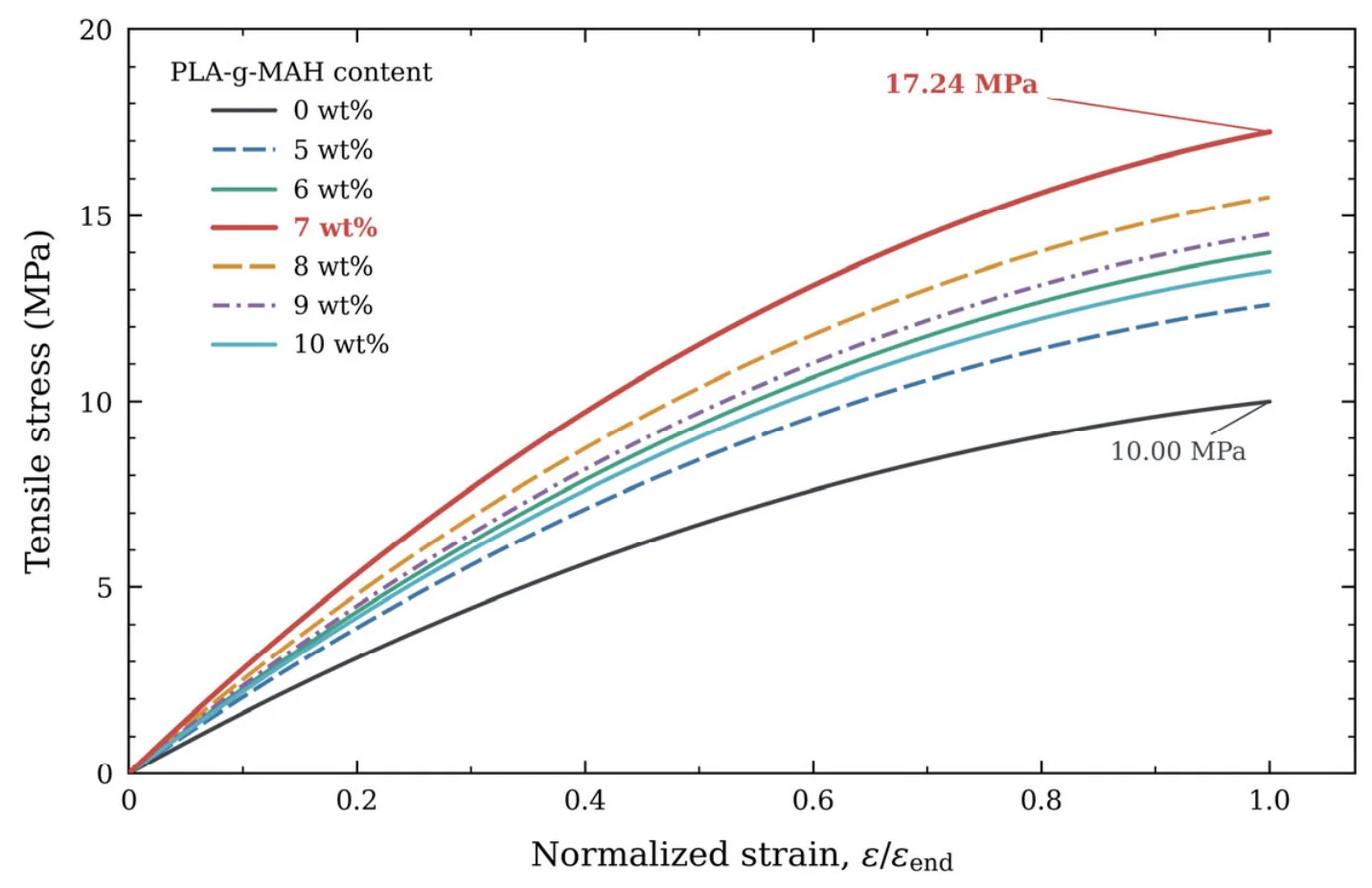
Schematic tensile stress–strain curves of WS/PLA biocomposites with different PLA-g-MAH contents, plotted against normalized strain.

Figure 3 summarizes the tensile-strength trend of WS/PLA biocomposites with different PLA-g-MAH contents. The tensile strength increased from 10.00 MPa for the uncompatibilized composite to 17.24 MPa at 7 wt% PLA-g-MAH, corresponding to an improvement of approximately 72%. This enhancement is consistent with the more compact fibre–matrix interface observed by SEM, which favors effective stress transfer. Further increases in PLA-g-MAH content reduced the tensile strength, possibly owing to local compatibilizer enrichment and increased structural heterogeneity. These results suggest that an appropriate compatibilizer dosage is essential for improving interfacial reinforcement, with 7 wt% providing the highest tensile strength under the investigated conditions.

### 3.2. X-Ray Diffraction Analysis

**Figure 4:**
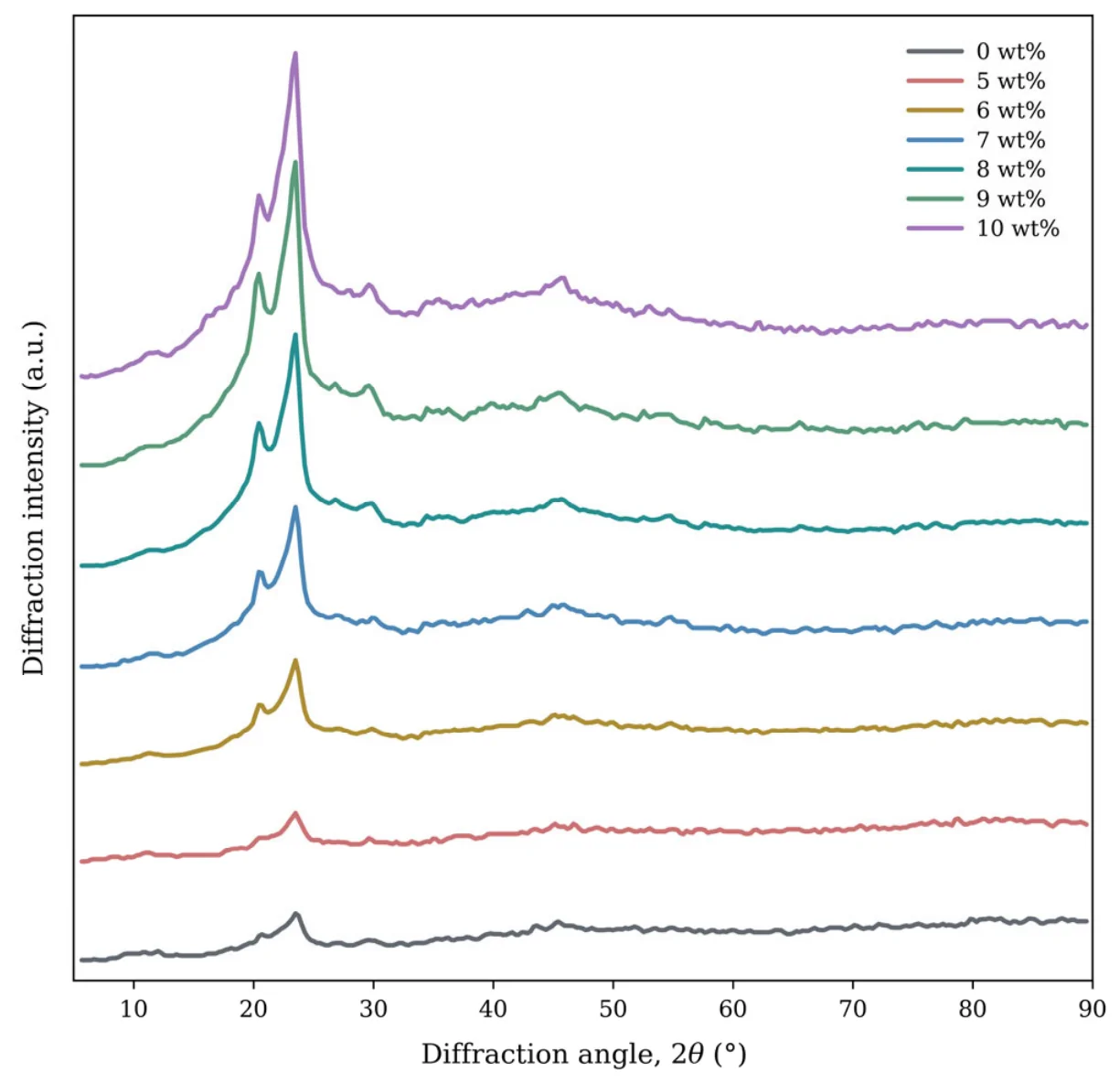
XRD patterns of WS/PLA biocomposites containing different PLA-g-MAH contents.

Figure 4 presents the XRD patterns of WS/PLA biocomposites containing different amounts of PLA-g-MAH. The diffraction profiles displayed characteristic peaks at approximately $2\theta =16.7^{\circ }$, $19.1^{\circ }$, and $22.3^{\circ }$. The position of these characteristic peaks remained broadly similar across the formulations, whereas their intensity and peak width changed with PLA-g-MAH content.

At 5 wt% PLA-g-MAH, the diffraction peaks became sharper than those of the uncompatibilized sample. The peak intensity increased further at 7 wt%, particularly near $2\theta =16.7^{\circ }$ and $22.3^{\circ }$, suggesting a more ordered crystalline organization of the PLA phase under this compatibilization condition. In contrast, when the PLA-g-MAH content was increased to approximately 9–10 wt%, the diffraction intensity decreased slightly and the peaks broadened, indicating a reduction in crystalline order relative to the 7 wt% formulation.

The XRD response therefore exhibits a trend similar to that observed in the mechanical tests. Moderate PLA-g-MAH addition may improve the local interfacial environment and facilitate more favorable packing or organization of PLA chains in the vicinity of the wheat straw surface. However, excessive PLA-g-MAH may disrupt this organization through local enrichment, dilution, or plasticization-like effects.

Importantly, the XRD results should be interpreted as evidence of changes in crystallization behaviour, rather than direct evidence of chemical bond formation. The observed correspondence between the strongest diffraction response and the optimum mechanical properties at 7 wt% supports a relationship between interfacial organization, crystalline structure, and macroscopic performance.

### 3.3. Interfacial Morphology

**Figure 5:**
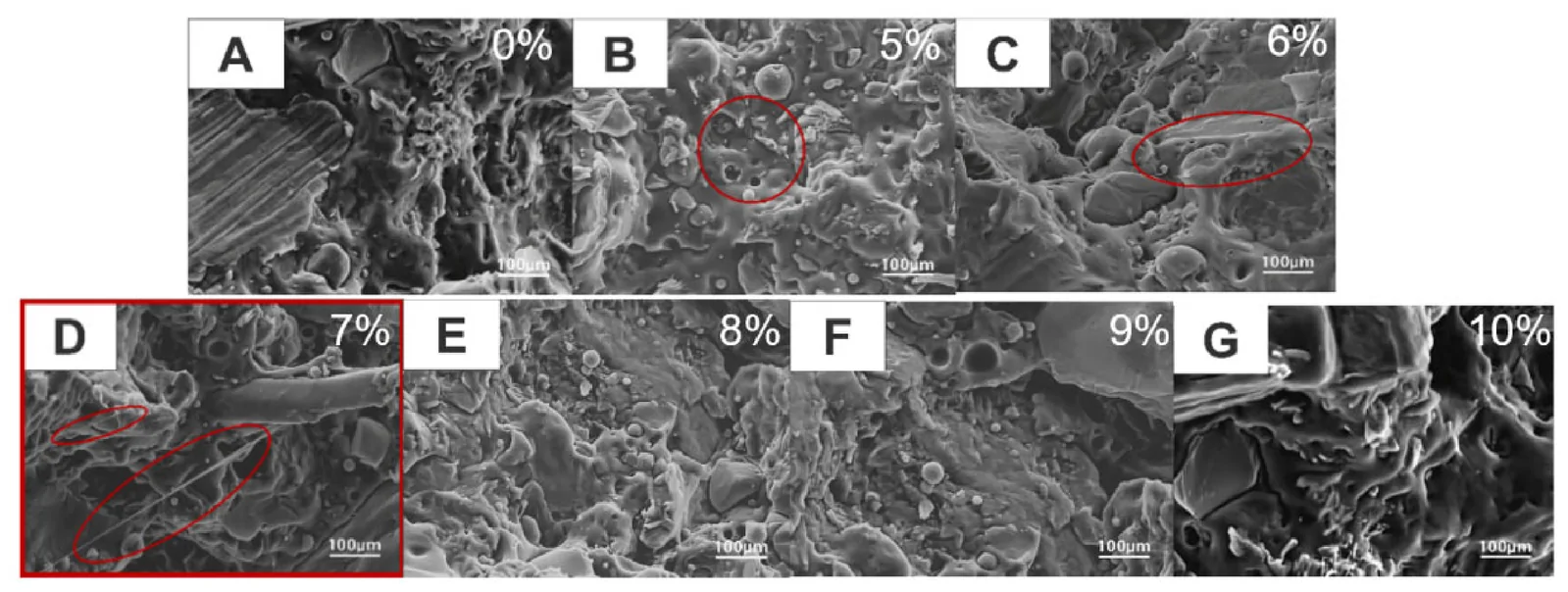
SEM images of fracture surfaces of WS/PLA biocomposites with different PLA-g-MAH contents: (**A**) 0 wt%; (**B**) 5 wt%; (**C**) 6 wt%; (**D**) 7 wt%; (**E**) 8 wt%; (**F**) 9 wt%; and (**G**) 10 wt%. The red circles highlight representative interfacial features, including residual gaps/voids at 5–6 wt% PLA-g-MAH and local fibrillation/drawing features associated with improved fibre–matrix adhesion at 7 wt%. The red box highlights the 7 wt% PLA-g-MAH formulation, which exhibited the most compact interfacial morphology.

SEM observations provide direct morphological evidence for the effect of PLA-g-MAH on the fibre–matrix interface. Representative fracture surfaces are shown in Figure 5.

In the uncompatibilized WS/PLA composite, distinct boundaries were visible between wheat straw particles and the PLA matrix. Numerous interfacial gaps and pores were present, and partial detachment of straw particles from the surrounding matrix was observed. These features indicate insufficient fibre wetting and weak interfacial adhesion, which are consistent with the relatively low mechanical performance of the control material.

With increasing PLA-g-MAH content, the fibre–matrix boundary became progressively less distinct and the number of visible gaps and voids decreased. At approximately 7 wt%, the fracture surface exhibited the most compact interfacial morphology, with wheat straw particles more tightly embedded in the PLA matrix and fewer obvious interfacial defects. Local fibrillation and drawing features were also observed, suggesting more effective deformation and load transfer across the interface. These representative features are highlighted by the red circles in Figure 5.

The improved morphology at 7 wt% provides a microstructural explanation for the simultaneous enhancement of tensile and impact performance. A more continuous interface enables stresses generated in the PLA phase to be transferred more effectively to the lignocellulosic reinforcement, while reduced interfacial porosity limits premature crack initiation.

When the PLA-g-MAH content was increased beyond the optimum level, localized signs of interfacial heterogeneity became apparent. SEM observations revealed slight phase separation and the formation of compatibilizer-rich regions at higher PLA-g-MAH contents, accompanied by reduced interfacial uniformity. These morphological changes were consistent with the decline in mechanical properties observed at excessive PLA-g-MAH dosages.

It should be emphasized that SEM confirms improved interfacial morphology and adhesion, but does not by itself demonstrate formation of specific covalent bonds. Therefore, the morphological results are more appropriately interpreted as being consistent with enhanced physical and possible chemical interactions induced by PLA-g-MAH.

### 3.4. Density

**Figure 6:**
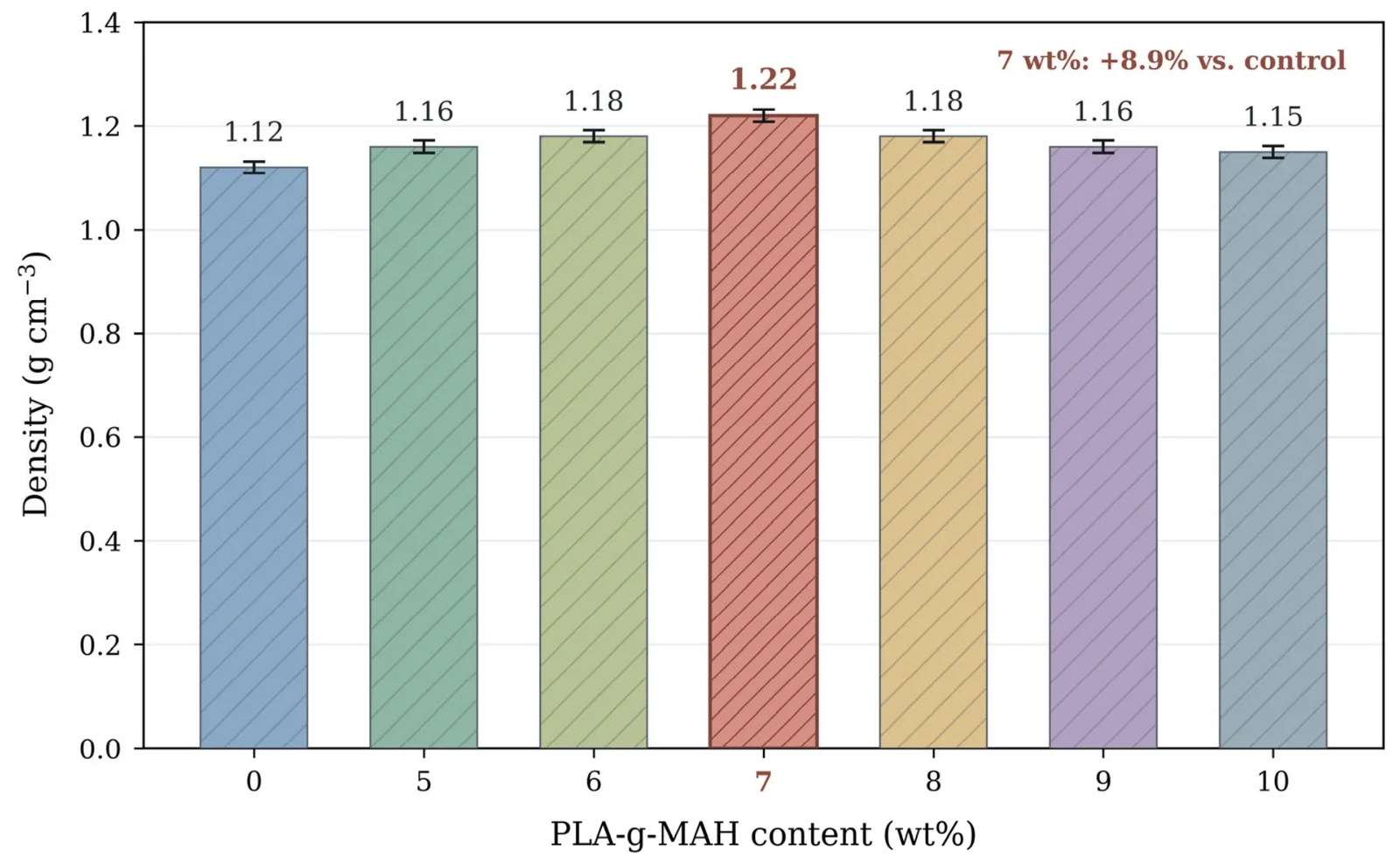
Effect of PLA-g-MAH content on the density of WS/PLA biocomposites. Data are presented as mean ± SD (n = 3). Different colors distinguish the PLA-g-MAH contents, and the numerical values above the bars indicate the mean density values. The annotation “7 wt%: +8.9% vs. control” indicates the percentage increase in density relative to the uncompatibilized control.

The density of the WS/PLA biocomposites initially increased with increasing PLA-g-MAH content and subsequently decreased slightly at higher dosages. As shown in Figure 6, the maximum density of approximately 1.22 g/cm^3^ was obtained at 7 wt% PLA-g-MAH.

The increase in density from the uncompatibilized condition to approximately 7 wt% PLA-g-MAH is consistent with improved consolidation of the fibre–matrix interface. Enhanced compatibility can reduce interfacial gaps and internal void volume, resulting in a more compact composite structure. This interpretation is strongly supported by the SEM observations, which showed the most continuous and compact interface at approximately the same PLA-g-MAH dosage.

At higher compatibilizer contents, the slight decline in density may reflect increasing heterogeneity within the interfacial region. Excess PLA-g-MAH that is not effectively accommodated at the fibre–matrix interface may form localized compatibilizer-rich domains, reducing structural uniformity and partially offsetting the densification achieved at moderate addition levels. The similarity between the density and mechanical-property trends further suggests that internal compactness is closely related to effective stress transfer in this composite system.

### 3.5. Water Absorption

**Figure 7:**
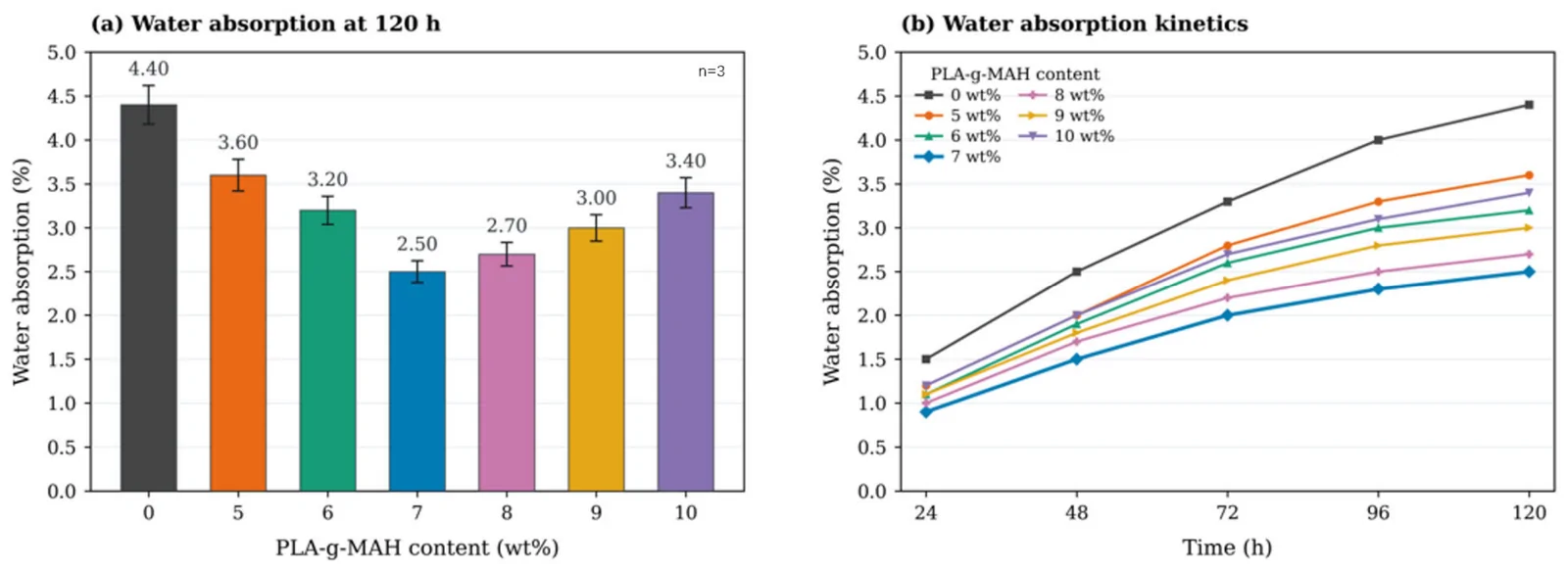
Water absorption behaviour of WS/PLA biocomposites with different PLA-g-MAH contents: (**a**) water absorption at 120 h as a function of PLA-g-MAH content; (**b**) water absorption kinetics over 24–120 h. Data in panel (**a**) are presented as mean ± SD (*n* = 3).

As shown in Figure 7a, the water absorption at 120 h exhibited a non-monotonic dependence on PLA-g-MAH content. Water absorption decreased progressively from 4.40% for the uncompatibilized composite to a minimum of 2.50% at 7 wt% PLA-g-MAH, corresponding to a reduction of approximately 43.2%. With further increases in PLA-g-MAH content, water absorption increased to 2.70%, 3.00%, and 3.40% at 8, 9, and 10 wt%, respectively. These results indicate that 7 wt% PLA-g-MAH provided the greatest improvement in resistance to water uptake among the investigated formulations.

Figure 7b further shows the evolution of water absorption during immersion from 24 to 120 h. Water absorption increased progressively with immersion time for all formulations, although the rate of increase gradually decreased at longer immersion times. The uncompabilized composite consistently exhibited the highest water absorption, whereas the composite containing 7 wt% PLA-g-MAH maintained the lowest values throughout the immersion period. Overall, the results suggest that an appropriate PLA-g-MAH content can reduce moisture penetration in WS/PLA biocomposites, while excessive compatibilizer addition partially diminishes this beneficial effect.

The reduced water absorption at moderate PLA-g-MAH contents can be primarily attributed to improved fibre–matrix compatibility and interfacial compactness. PLA-g-MAH promotes wetting and adhesion between the hydrophilic wheat straw and the comparatively hydrophobic PLA matrix, thereby reducing interfacial gaps, pores, and microcracks that can serve as preferential pathways for water transport. This explanation is consistent with the SEM observations, which showed fewer interfacial defects and a more continuous fibre–matrix interface at 7 wt% PLA-g-MAH. In addition, possible interactions between the maleic anhydride groups of PLA-g-MAH and hydroxyl-containing sites on wheat straw may reduce the effective availability of polar sites for water adsorption.

When the PLA-g-MAH content exceeded 7 wt%, the increase in water absorption may have resulted from the formation of compatibilizer-rich regions and reduced interfacial uniformity. Such heterogeneity can generate local microvoids and reopen pathways for moisture penetration. The water-absorption results therefore agree with the mechanical, density, XRD, and SEM observations, collectively confirming that approximately 7 wt% PLA-g-MAH provides the most favourable interfacial structure and moisture resistance under the investigated conditions.

### 3.6. Thermal Properties

#### 3.6.1. Thermal Stability

**Figure 8:**
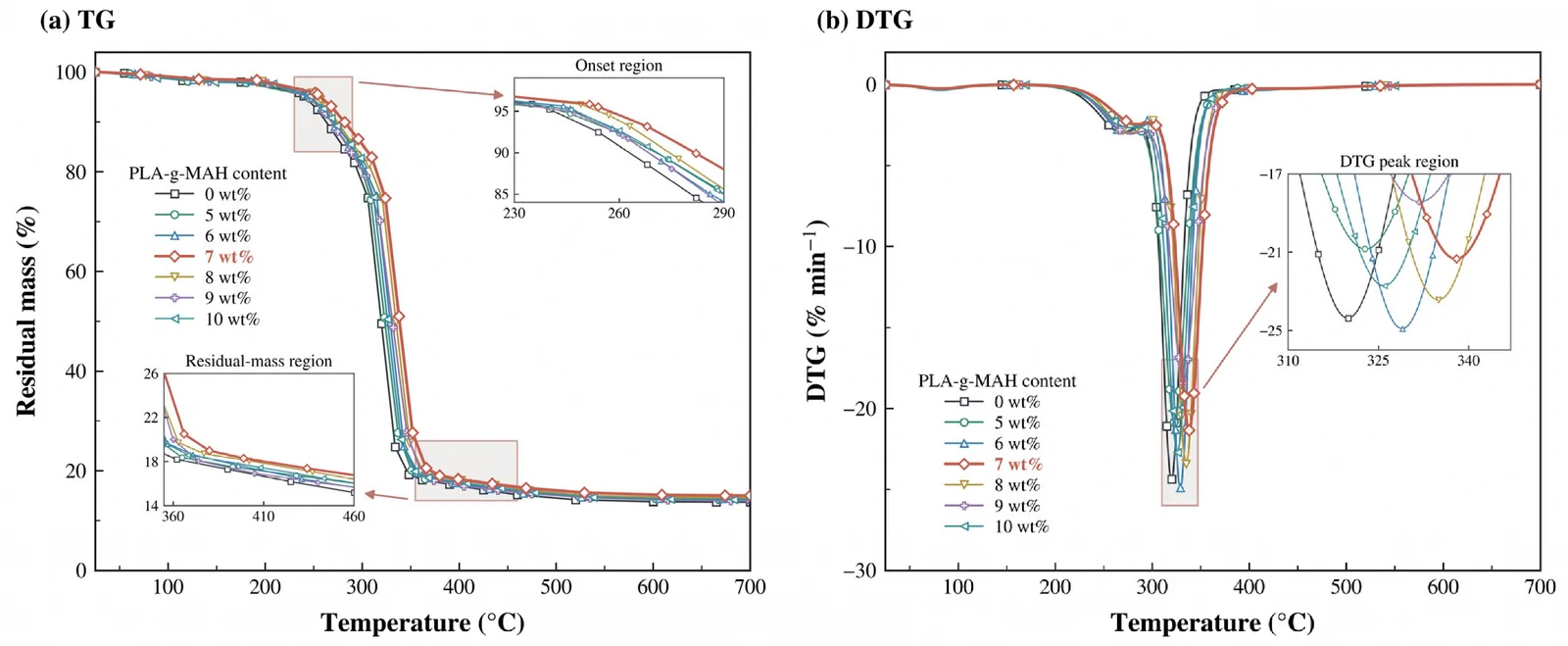
Thermal degradation behaviour of WS/PLA biocomposites with different PLA-g-MAH contents: (**a**) TG curves and (**b**) DTG curves.

Figure 8 shows the TG and DTG curves of WS/PLA biocomposites containing different PLA-g-MAH contents. All composites exhibited similar overall thermal degradation profiles, indicating that the incorporation of PLA-g-MAH did not fundamentally change the degradation mechanism. However, the main degradation region gradually shifted toward higher temperatures as the PLA-g-MAH content increased up to 7 wt%. The 7 wt% PLA-g-MAH composite showed the highest onset and maximum degradation temperatures, together with relatively higher residual mass, indicating improved thermal stability. When the PLA-g-MAH content was further increased to 8–10 wt%, this improvement was reduced. This behaviour may be attributed to improved interfacial compatibility between WS and the PLA matrix at an appropriate PLA-g-MAH content, which can restrict thermal degradation and improve the thermal resistance of the composite.

#### 3.6.2. Melting and Crystallization Behaviour

**Figure 9:**
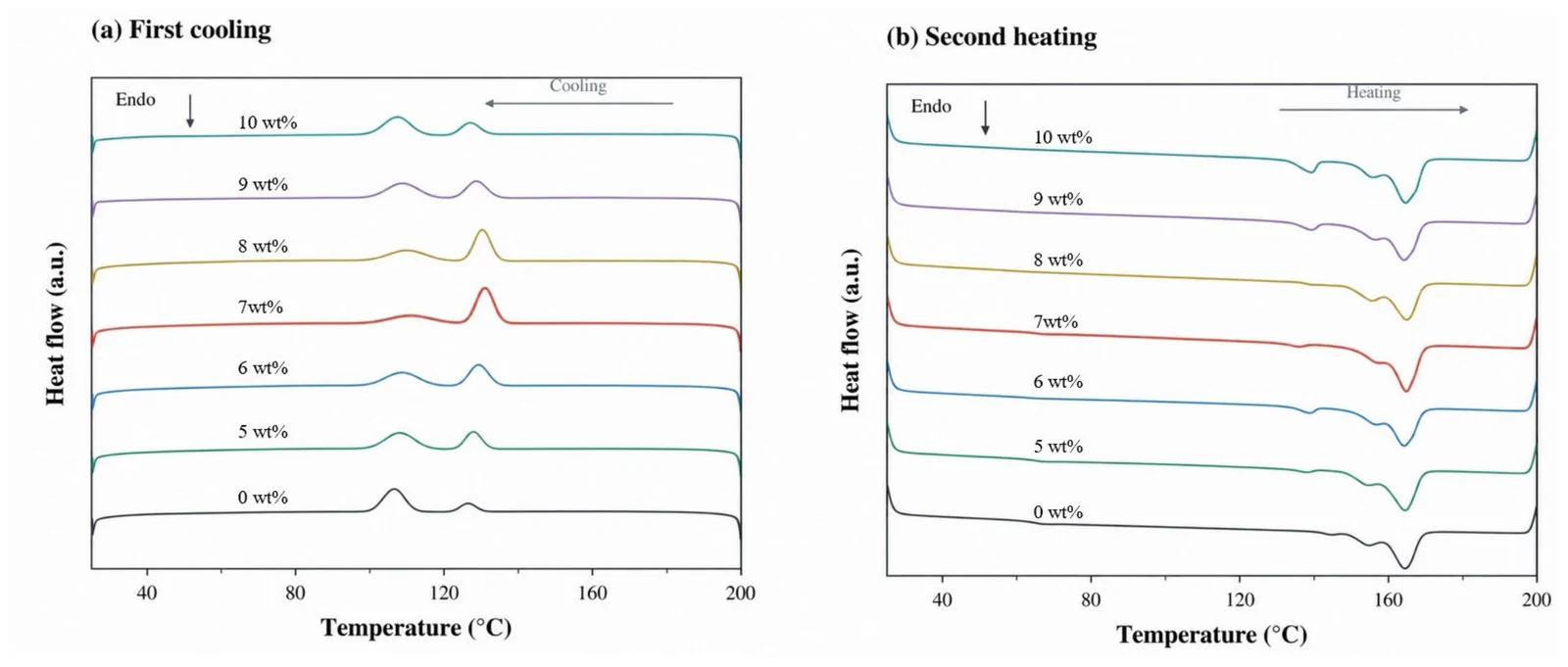
DSC thermograms of WS/PLA biocomposites with different PLA-g-MAH contents: (**a**) first-cooling curves and (**b**) second-heating curves.

Figure 9 presents the DSC first-cooling and second-heating curves of the WS/PLA biocomposites. During cooling, the incorporation of PLA-g-MAH changed both the position and intensity of the crystallization peaks, indicating that PLA-g-MAH affected the crystallization behaviour of the PLA phase. The 7 wt% PLA-g-MAH composite exhibited a more pronounced high-temperature crystallization peak, suggesting a favorable effect on crystallization. During the second-heating cycle, changes in the melting peaks were also observed with increasing PLA-g-MAH content, indicating that the compatibilizer influenced the crystalline organization and melting behaviour of PLA. The effect was not continuously enhanced at higher PLA-g-MAH contents, suggesting that an appropriate compatibilizer content is more favorable for regulating the crystalline structure of the WS/PLA composites.

### 3.7. Integrated Structure–Property Relationship

The mechanical, physical, XRD, SEM, TGA, and DSC results collectively demonstrate a clear non-monotonic, dosage-dependent response of WS/PLA biocomposites to PLA-g-MAH addition. The evolution of composite performance can be interpreted in terms of three interfacial states: insufficient compatibilization at low PLA-g-MAH contents, optimal interfacial modification at approximately 7 wt%, and over-compatibilization at higher dosages. At low PLA-g-MAH contents, the amount of compatibilizer is insufficient to effectively modify the extensive interface between wheat straw and the PLA matrix. Consequently, incomplete fibre wetting, interfacial gaps, voids, and partial fibre pull-out remain evident. These defects interrupt stress transfer across the fibre–matrix interface and provide preferential pathways for moisture penetration. The relatively low structural compactness under these conditions is therefore consistent with the lower mechanical performance, lower density, and higher water absorption observed experimentally. The XRD results further suggest that the PLA phase exhibits comparatively less favorable crystalline organization under insufficient compatibilization.

When the PLA-g-MAH content increases to approximately 7 wt%, a substantially more continuous and compact fibre–matrix interface is obtained. SEM observations show fewer interfacial voids and reduced fibre pull-out, indicating improved embedding of wheat straw within the PLA matrix. Such interfacial improvement facilitates more efficient stress transfer from the PLA matrix to the lignocellulosic reinforcement and simultaneously reduces the continuity of pathways available for water penetration. The increase in composite density at this dosage further supports the formation of a more compact internal structure. In parallel, the stronger and sharper XRD response suggests more favorable crystalline organization of the PLA phase. The TGA results further showed improved thermal stability at this dosage, while the DSC results indicated favorable changes in the crystallization and melting behaviour of the PLA phase. As a result of these mutually reinforcing structural changes, the 7 wt% formulation exhibited the most favorable overall performance, with a tensile strength of 17.24 MPa, an impact strength of 15.56 kJ/m^2^, a density of approximately 1.22 g/cm^3^, and a reduction in water absorption of approximately 43.2% relative to the uncompatibilized control.

Further increasing the PLA-g-MAH content to 8–10 wt% did not produce additional improvement. Instead, the simultaneous decline in mechanical properties and density, together with the increase in water absorption and the weakening and broadening of the XRD response, indicates that excessive compatibilizer disrupts the optimum interfacial state. At these higher dosages, PLA-g-MAH may accumulate locally and form compatibilizer-rich or compositionally heterogeneous interfacial regions. Such heterogeneity can reduce structural uniformity, decrease stress-transfer efficiency, promote the formation of local microvoids, and partially reopen pathways for moisture transport. Excessive PLA-g-MAH may also interfere with the local organization of PLA chains, which is consistent with the less favorable XRD response observed at higher compatibilizer contents.

Overall, the structure–property relationship can therefore be summarized as follows: PLA-g-MAH dosage governs the interfacial state of the WS/PLA composite, which in turn affects internal compactness and crystalline organization, controls the efficiency of stress transfer and moisture transport, and ultimately determines the macroscopic mechanical and physical properties. Under the formulation and hot-pressing conditions investigated in this study, approximately 7 wt% PLA-g-MAH provides the most favorable balance between interfacial modification and preservation of structural uniformity.

It should be noted that the proposed compatibilization mechanism is inferred primarily from the combined mechanical, physical, XRD, and SEM evidence. Although these results consistently support enhanced interfacial interactions after PLA-g-MAH addition, they do not directly confirm the formation of specific chemical bonds between PLA-g-MAH and wheat straw. Direct identification of such interactions would require complementary spectroscopic characterization, such as FTIR or XPS. In addition, the optimum dosage identified in the present study is specific to the wheat straw content and processing conditions employed and should therefore not be regarded as universally applicable to other fibre loadings or processing routes.

## 4. Conclusions

This study systematically investigated the effect of PLA-g-MAH content on the mechanical, physical, crystalline, thermal, and interfacial properties of wheat straw/poly(lactic acid) (WS/PLA) biocomposites containing a fixed wheat straw content of 50 wt%. The results demonstrate that the effect of PLA-g-MAH was strongly dosage-dependent rather than continuously increasing with compatibilizer concentration.

Mechanical performance improved progressively as the PLA-g-MAH content increased toward 7 wt%. At this dosage, the tensile strength reached 17.24 MPa and the impact strength reached 15.56 kJ/m^2^, corresponding to increases of approximately 72% and 55%, respectively, compared with the uncompatibilized control. Further increases in PLA-g-MAH content resulted in a decline in mechanical performance, indicating the occurrence of an optimum compatibilization range.

The physical-property results showed a consistent trend. The composite density reached a maximum of approximately 1.22 g/cm^3^ at 7 wt% PLA-g-MAH, indicating improved structural compactness, while the water absorption decreased by approximately 43.2% compared with the uncompatibilized composite. These findings suggest that appropriate compatibilizer addition not only improves load transfer but also reduces interfacial pathways for moisture penetration [36,37].

XRD analysis showed that moderate PLA-g-MAH addition altered the crystallization behaviour of the PLA phase, with the strongest and sharpest diffraction response observed around 7 wt%. At higher compatibilizer contents, peak intensity decreased and peak broadening increased, indicating a reduction in crystalline order. SEM observations further confirmed that the 7 wt% formulation exhibited the most continuous and compact fibre–matrix interface, characterized by fewer interfacial voids, reduced fibre pull-out, and improved embedding of wheat straw within the PLA matrix. Excessive PLA-g-MAH addition resulted in localized interfacial heterogeneity and signs of compatibilizer-rich regions. Thermal analyses further showed that PLA-g-MAH affected the thermal stability, crystallization, and melting behaviour of the composites, supporting the dosage-dependent compatibilization effect.

Overall, the results establish a consistent structure–property relationship in which an appropriate amount of PLA-g-MAH improves fibre–matrix compatibility, reduces interfacial defects, enhances composite compactness and crystalline organization, promotes more efficient stress transfer, and restricts moisture penetration. Under the formulation and processing conditions investigated, approximately 7 wt% PLA-g-MAH was identified as the optimum compatibilizer dosage for 50 wt% WS/PLA biocomposites. From a practical perspective, the approximately 7 wt% PLA-g-MAH formulation provides a useful reference for producing wheat-straw/PLA biocomposites with improved mechanical integrity and moisture resistance. These characteristics indicate potential for the development of bio-based panels and molded products where improved mechanical performance and reduced moisture uptake are desirable. However, the optimum dosage identified in this study is specific to the fixed 50 wt% wheat-straw loading and the hot-pressing route employed. Although the combined mechanical, physical, morphological, crystalline, and thermal results support improved interfacial compatibility, the specific chemical interactions between PLA-g-MAH and wheat straw were not directly confirmed. Future studies should therefore investigate different fibre loadings and processing conditions and incorporate complementary spectroscopic analyses, such as FTIR or XPS, to further clarify the compatibilization mechanism and long-term durability.

## Figures and Tables

**Table 1:** Formulations of WS/PLA biocomposites with different PLA-g-MAH contents.

PLA-g-MAH Content	PLA (g)	WS (g)	PLA-g-MAH (g)	Total Mass (g)
0 wt%	4.00	4.00	0.00	8.00
5 wt%	3.60	4.00	0.40	8.00
6 wt%	3.52	4.00	0.48	8.00
7 wt%	3.44	4.00	0.56	8.00
8 wt%	3.36	4.00	0.64	8.00
9 wt%	3.28	4.00	0.72	8.00
10 wt%	3.20	4.00	0.80	8.00

**Table 2:** Mechanical properties of WS/PLA biocomposites with different PLA-g-MAH contents.

PLA-g-MAH Content (wt%)	Density (g/cm^3^)	Tensile Strength (MPa)	Flexural Strength (MPa)	Impact Strength (kJ/m^2^)
0	1.12 ± 0.011	10.9 ± 0.55	18.2 ± 0.91	10.0 ± 0.5
5	1.16 ± 0.013	12.7 ± 0.62	20.6 ± 1.02	12.2 ± 0.60
6	1.18 ± 0.012	14.0 ± 0.69	23.5 ± 1.16	13.0 ± 0.64
7	1.22 ± 0.011	17.0 ± 0.82	25.9 ± 1.29	15.8 ± 0.78
8	1.18 ± 0.012	15.6 ± 0.77	24.2 ± 1.22	14.8 ± 0.72
9	1.16 ± 0.012	14.5 ± 0.71	22.8 ± 1.13	13.2 ± 0.63
10	1.15 ± 0.013	13.6 ± 0.64	20.2 ± 1.01	12.5 ± 0.61

## Data Availability

The data supporting the findings of this study are available from the corresponding author upon reasonable request.
